# Association of 24 h Pulse Pressure Derived from Ambulatory Blood Pressure Monitoring with All-Cause and Cardiovascular Mortality in Patients Undergoing Peritoneal Dialysis: A Prospective Cohort Study

**DOI:** 10.3390/jcm15197554

**Published:** 2026-09-29

**Authors:** Jing Yu, Rui Yang, Xi Xia, Jianwen Yu, Yagui Qiu, Jianbo Li, Xiao Yang, Haiping Mao, Chunyan Yi, Qinghua Liu, Wei Chen

**Affiliations:** 1 Department of Nephrology, the First Affiliated Hospital, Sun Yat-Sen University, 58th, Zhongshan Road II, Guangzhou 510080, Chinaxiax29@mail.sysu.edu.cn (X.X.);; 2 Key Laboratory of Nephrology, Ministry of Health and Guangdong Province, Guangzhou 510080, China

**Keywords:** 24 h pulse pressure, ambulatory blood pressure monitoring, peritoneal dialysis, cardiovascular mortality, arterial stiffness

## Abstract

**Background/Objectives:** Pulse pressure (PP), an indirect hemodynamic marker reflecting the pulsatile component of blood pressure, has been linked to adverse outcomes in dialysis patients. Whether 24 h PP (24 h PP) derived from ambulatory BP monitoring (ABPM) is independently associated with all-cause and cardiovascular (CV) mortality in peritoneal dialysis (PD) patients remains unclear. **Methods:** This sub-cohort, nested within a single-center prospective PD database, enrolled 165 patients with baseline 24 h ABPM. The 24 h PP was analyzed continuously and in tertiles. All-cause mortality was assessed by Cox regression and CV mortality by Fine–Gray competing-risk modeling. The median follow-up was 47.4 months, counted from the date of ABPM completion. **Results:** During follow-up, 48 patients (29.1%) died, including 28 CV deaths (58.3% of all deaths). Per 1-SD increment in 24 h PP, the adjusted hazard ratio (HR) for all-cause mortality was 1.39 (95% confidence interval [CI] 1.03–1.87; *p* = 0.032), and the adjusted subdistribution hazard ratio (SHR) for CV mortality was 1.44 (95% CI 1.06–1.94; *p* = 0.018). The highest tertile showed elevated risks of all-cause (HR 3.47; 95% CI 1.12–10.70; *p* = 0.031) and CV mortality (SHR 5.11; 95% CI 1.32–19.79; *p* = 0.018) versus the lowest, persisting after adjustment for office PP. **Conclusions:** Elevated ABPM-derived 24 h PP is independently associated with increased all-cause and CV mortality in PD patients after adjustment for office PP.

## 1. Introduction

Cardiovascular (CV) mortality remains the leading cause of death in end-stage renal disease (ESRD), accounting for over half of all fatalities in this population [1]. Hypertension is highly prevalent among dialysis patients and constitutes a major modifiable contributor to this excess risk, yet adequate blood pressure (BP) control is achieved in only a minority of cases [2].

Office and peridialytic BP measurements are complicated by reverse epidemiology, in which lower BP tends to coincide with poorer survival because of confounding by frailty, malnutrition, and advanced comorbidity [3,4]. Moreover, single-occasion readings may miss masked hypertension, which affects roughly one fifth of peritoneal dialysis (PD) patients, and fail to capture diurnal BP patterns that carry independent prognostic relevance [5]. Twenty-four-hour ambulatory BP monitoring (24 h ABPM), now widely regarded as the reference-standard method for diagnosing hypertension in dialysis populations, yields indices that correlate more closely with target-organ damage and survival than do office readings [6,7]. A 2025 meta-analysis of 28,016 PD patients reported a consistent linear association between higher ambulatory BP and both all-cause and CV mortality [8]. Nevertheless, most ABPM studies in dialysis have focused on systolic, diastolic, and nocturnal BP levels, while the significance of 24 h pulse pressure (24 h PP) has received comparatively little attention.

PP reflects the pulsatile component of hemodynamic load and is influenced by arterial compliance, stroke volume, cardiac function, age, and volume status; it should therefore be regarded as an indirect hemodynamic marker rather than a direct measure of arterial stiffness such as pulse-wave velocity (PWV), although it is commonly used as a surrogate of large-artery stiffness in epidemiological research [9]. In community-dwelling populations, widened PP has been independently associated with coronary events, stroke, heart failure, and death [10,11]. In hemodialysis (HD) patients, early ABPM work by Amar et al. demonstrated that 24 h PP was strongly associated with mortality [12], and subsequent cohorts have corroborated this finding [13,14]. In PD patients, by contrast, available evidence has come almost exclusively from office or home BP measurements: higher home PP predicted death and CV events in one cohort [15], and office PP above 60 mmHg was associated with increased mortality in a Chinese PD cohort [16]. Evidence specifically examining ABPM-derived 24 h PP in PD patients, particularly after adjustment for office BP and accounting for competing risks, remains limited. We therefore conducted this prospective cohort study to examine the association of ABPM-derived 24 h PP with all-cause and CV mortality in PD patients.

## 2. Materials and Methods

### 2.1. Study Population

Data for this analysis were drawn from a prospectively maintained PD cohort database at the First Affiliated Hospital of Sun Yat-sen University. This ABPM-specific sub-study enrolled eligible patients between 1 January 2017 and 30 June 2021. Inclusion criteria were age ≥18 years, PD catheter insertion at our center, expected PD duration ≥3 months, written informed consent, and a technically satisfactory 24 h ABPM recording. Patients were excluded if they had undergone prior kidney transplantation, had been maintained on HD for more than 3 months, had active malignancy, or had incomplete ABPM data. The completion date of ABPM served as the baseline index date, and survival follow-up was initiated from this date to prevent immortal-time bias. This ABPM sub-study was embedded in our prospectively maintained PD registry, and the primary analysis was prespecified at the time of ABPM enrollment. Of 565 incident PD patients treated during the enrollment period, 187 agreed to undergo 24 h ABPM; after excluding 22 patients, 165 with valid recordings were included (voluntary participation rate, approximately 33%). All participants were followed until 31 December 2025. The study was approved by the institutional ethics committee and conducted in accordance with the Declaration of Helsinki.

### 2.2. 24 h Ambulatory Blood Pressure Monitoring

ABPM was performed using the validated TM-2430 oscillometric upper-arm monitor (A&D Company, Tokyo, Japan) [17], with cuff size selected according to the participant’s mid-arm circumference. The device was fitted in the morning at the outpatient PD clinic and removed 24 h later; during this period patients continued their prescribed PD exchange schedule without modification, and no patient underwent a dwell exchange, peritoneal equilibration test, or acute ultrafiltration adjustment on the recording day. Participants were instructed to maintain their usual daily activities, avoid strenuous exercise, and keep the arm still during each automated inflation. Readings were programmed every 20 min during the day and every 30 min at night. Daytime (07:00–23:00) and nighttime (23:00–07:00) were defined by fixed clock intervals; individual sleep/wake diaries were not collected. A recording was considered valid when at least 70% of scheduled measurements were obtained and no continuous gap exceeded 3 h. BP target attainment was defined as 24 h average BP < 130/80 mmHg, daytime < 135/85 mmHg, and nighttime < 120/70 mmHg [18]. Participants were not informed of their ABPM results during follow-up.

### 2.3. Office Blood Pressure Measurement

Office BP was measured at ABPM placement and retrieval by a single dedicated PD nurse using a validated oscillometric sphygmomanometer; patients sat quietly for 5–10 min before each reading, and no fasting was required. The mean of the two visits was used, and office BP <140/90 mmHg was considered target attainment [18].

### 2.4. Baseline Data and Laboratory Assessments

Demographic characteristics, comorbid conditions, and medication histories were abstracted from electronic medical records at the time of initial admission. Diabetes was defined by standard diagnostic criteria or ongoing hypoglycemic therapy; prior CV events comprised angina pectoris, myocardial infarction, heart failure, coronary revascularization, and cerebrovascular accident [19]. Hypertension was defined as office BP ≥ 140/90 mmHg or regular antihypertensive use.

Routine laboratory tests, including hemoglobin, serum albumin (ALB), high-sensitivity C-reactive protein (Hs-CRP), serum creatinine, uric acid, and a full lipid panel, were performed within the first 3 months after PD initiation, according to the hospital’s standardized laboratory protocols. Estimated glomerular filtration rate (eGFR) was calculated using the Chronic Kidney Disease Epidemiology Collaboration (CKD-EPI) equation [20]. Baseline antihypertensive drug class and number of agents were recorded.

### 2.5. Follow-Up and Outcome Ascertainment

The primary outcome was all-cause mortality, and the secondary outcome was CV mortality, defined as death attributable to cardiac or cerebrovascular causes, including acute myocardial infarction, sudden cardiac death, ischemic heart failure, stroke, peripheral arterial disease, arrhythmia, valvular or pericardial disease, unexplained sudden death, and pulmonary embolism [21]. All deaths were independently adjudicated by at least two board-certified attending physicians using death certificates, inpatient records, coronary or cerebral imaging, and laboratory results; disagreements were resolved by consensus. For out-of-hospital deaths without formal medical documentation, telephone interviews with next of kin were conducted to ascertain the terminal clinical course [22,23].

A dedicated follow-up team of nephrologists and trained nurses maintained monthly telephone or in-person contact with each patient and quarterly outpatient reviews [22]. Person-time was calculated from the ABPM completion date to death, censoring, or 31 December 2025. Patients were censored at the end of follow-up or earlier if they transferred to HD (*n* = 14), underwent kidney transplantation (*n* = 20), moved to another center, or were lost to follow-up; these modality changes were treated as censoring rather than competing events.

### 2.6. Statistical Analysis

Participants were divided into tertiles of baseline 24 h PP: T1 (<48 mmHg), T2 (48–54 mmHg), and T3 (>54 mmHg), with T1 as the reference. These data-derived cut-offs are reported as secondary descriptive analyses; the primary exposure was 24 h PP modeled continuously per 1-SD increment. Continuous variables are presented as mean ± standard deviation (SD) or median (interquartile range, IQR), and categorical variables as counts (percentages); between-group comparisons used ANOVA, the Kruskal–Wallis test, or chi-square as appropriate.

Univariable logistic regression identified factors associated with elevated 24 h PP. Based on prior studies and pathophysiology, prespecified clinically relevant candidates—age, sex, smoking, body mass index (BMI), diabetes, prior CV events, eGFR, albumin, hemoglobin, and Hs-CRP—together with any variable reaching univariable *p* < 0.20—were entered into multivariable logistic models [24]. Odds ratios (ORs) with 95% confidence intervals (CIs) are reported.

Kaplan–Meier curves with the log-rank test compared all-cause mortality across tertiles, while cumulative incidence functions (CIFs) with the Fine–Gray test compared CV mortality, accounting for competing non-CV death. Restricted cubic splines with three knots at the 10th, 50th, and 90th percentiles of 24 h PP (44, 54, and 71 mmHg) depicted the continuous dose–response. Splines were plotted over the 5th–95th percentile range (42–76 mmHg), with a rug plot showing the distribution of individual observations. A formal *p* value for the nonlinear spline term was reported to test for departure from linearity.

Multivariable Cox regression estimated hazard ratios (HRs) for all-cause mortality, and Fine–Gray regression estimated subdistribution hazard ratios (SHRs) for CV mortality. Three nested models were constructed: Model 1 adjusted for age and sex; Model 2 added diabetes, prior CV events, ALB, and uric acid; Model 3 further incorporated office PP. Covariates were selected a priori on clinical grounds rather than data-driven stepwise selection. A parsimonious sensitivity model containing only age, sex, diabetes, and prior CV events was also fitted, along with a cause-specific Cox model for CV mortality and 1000 bootstrap resamples. The proportional hazards assumption was evaluated using Schoenfeld residuals for the Cox model and Schoenfeld-type residuals for the Fine–Gray model. Complete-case analysis was used throughout; no missing data were observed among model covariates. All 165 participants were included in each multivariable model. A two-sided *p*-value < 0.05 was considered statistically significant. All analyses were performed using SPSS version 26.0 and Stata version 14.0.

## 3. Results

### 3.1. Baseline Characteristics of the Study Cohort

The final analytic cohort comprised 165 patients undergoing PD, categorized by 24 h PP into T1 (*n* = 55), T2 (*n* = 52), and T3 (*n* = 58) (Figure 1). The baseline clinical and biochemical characteristics are summarized in Table 1. Overall, the mean age was 42.4 ± 13.4 years, 58.2% were male, and 10.9% had diabetes, with a mean 24 h PP of 55.5 ± 10.6 mmHg. Compared with participants in the lower tertiles, those in T3 were older, more likely to have diabetes, and exhibited higher BMI, Hs-CRP, office systolic blood pressure (SBP)/diastolic blood pressure (DBP), and all ABPM-derived BP indices (24 h SBP, 24 h DBP, daytime SBP/DBP, and nighttime SBP).

During a median follow-up of 47.4 months, 48 patients (29.1%) died, of whom 28 (58.3% of all deaths) died from CV causes. All-cause mortality increased progressively across tertiles: 9.1% (5/55) in T1, 28.8% (15/52) in T2, and 48.3% (28/58) in T3; the corresponding CV mortality rates were 5.5%, 15.4%, and 29.3%, respectively. BP control was generally suboptimal in this cohort. Although 49.7% of patients achieved the office BP target (<140/90 mmHg), the proportions meeting ABPM-based targets were substantially lower: 21.2% for 24 h average BP, 33.3% for daytime BP, and only 9.7% for nighttime BP (Figure 2).

### 3.2. Factors Associated with Elevated 24 h PP

We next examined the baseline factors associated with elevated 24 h PP (Table 2). In univariable logistic regression, older age, diabetes, higher BMI, and lower eGFR were associated with T3 PP. After multivariable adjustment, older age (adjusted OR 1.08; 95% CI 1.03–1.12; *p* < 0.001), diabetes (adjusted OR 4.26; 95% CI 1.37–13.20; *p* = 0.012), and higher serum uric acid (adjusted OR 1.72; 95% CI 1.06–2.79; *p* = 0.028) remained independently associated with higher 24 h PP.

### 3.3. Associations Between 24 h PP and All-Cause and CV Mortality

Kaplan–Meier analysis demonstrated that patients in T3 had the lowest cumulative survival, with a statistically significant between-group difference in all-cause mortality (log-rank *p* = 0.011; Figure 3A). For CV mortality, cumulative incidence functions accounting for the competing risk of non-CV death showed the highest incidence in T3, with a significant across-tertile difference (Gray’s test *p* = 0.036; Figure 3B).

Restricted cubic spline modeling with three knots revealed an approximately linear positive relationship between 24 h PP and both all-cause (Figure 4A) and CV mortality (Figure 4B) after multivariable adjustment. Formal testing of the nonlinear spline term showed no significant departure from linearity for either all-cause (*p* for nonlinearity = 0.429) or CV mortality (*p* for nonlinearity = 0.761).

The multivariable regression results are presented in Table 3. In the fully adjusted model, each 1-SD increment in 24 h PP was independently associated with all-cause mortality (HR 1.39; 95% CI 1.03–1.87; *p* = 0.032) and CV mortality (SHR 1.44; 95% CI 1.06–1.94; *p* = 0.018). In categorical analyses, compared with T1, T3 yielded an all-cause HR of 3.47 (95% CI 1.12–10.70; *p* = 0.031) and a CV SHR of 5.11 (95% CI 1.32–19.79; *p* = 0.018); the corresponding T2 estimates were not statistically significant. Office PP and 24 h PP were moderately correlated (Pearson r = 0.54; *p* < 0.001), and all variance inflation factors in Model 3 were below 2.0, arguing against meaningful multicollinearity.

To assess robustness, we performed several sensitivity analyses. In the parsimonious model adjusted for age, sex, diabetes, and prior CV events only, the all-cause association remained significant (HR 1.31; 95% CI 1.01–1.70; *p* = 0.044), while the CV cause-specific estimate was directionally consistent but did not reach conventional significance (HR 1.24; 95% CI 0.90–1.71; *p* = 0.180). The cause-specific Cox model for CV mortality, treating non-CV deaths as censoring, yielded an HR of 1.45 (95% CI 1.00–2.09; *p* = 0.050) per 1-SD increment, closely concordant with the Fine–Gray SHR of 1.44. Schoenfeld residual tests showed no violation of the proportional hazards assumption for 24 h PP (all-cause mortality, *p* = 0.62; CV mortality, *p* = 0.55) or for sex, diabetes, prior CV events, albumin, uric acid, and office PP; age showed a borderline result in the all-cause model (*p* = 0.025), and repeating the analysis with age stratified by tertile yielded an essentially unchanged HR (1.40; 95% CI 1.03–1.90; *p* = 0.034). Finally, 1000 bootstrap resamples confirmed the stability of the primary all-cause association (median HR 1.42; 95% bootstrap CI 1.01–1.96).

## 4. Discussion

In this prospective cohort of 165 PD patients, elevated ABPM-derived 24 h PP was independently associated with both all-cause and CV mortality after adjustment for conventional risk factors and office PP.

Previous studies have consistently linked elevated PP to adverse outcomes in HD [12,13,14] and PD populations [8,15,16,25]. The most directly comparable prior work is that of Dai et al., who used 24 h ABPM in 260 PD patients and reported that ambulatory PP independently predicted all-cause and CV mortality [26]. Panuccio et al. further showed that home-measured PP predicted death and CV events [15], whereas Xie et al. observed a reverse L-shaped association between office PP and mortality [16]. The present study extends these observations in three respects. First, the association persisted after adjustment for office PP, indicating that ABPM-derived PP captures hemodynamic information not reflected in clinic readings, which are susceptible to white-coat effects and single-occasion variability. Second, CV mortality was analyzed using Fine–Gray competing-risk regression rather than treating non-CV death as censoring. Third, spline analysis suggested an approximately linear positive dose–response relationship, in contrast to the reverse L-shaped pattern reported for office PP [16]; this discrepancy may reflect confounding of office measurements by frailty and malnutrition, or differences in cohort characteristics, as our population was relatively young with a low comorbidity burden.

PP reflects the interplay between stroke volume and arterial compliance and serves as an indirect hemodynamic marker of large-artery stiffness [10,27]. In ESRD, arterial stiffening is driven largely by CKD–mineral and bone disorder (MBD) through medial vascular calcification [27,28], which widens PP, increases left ventricular afterload, and reduces diastolic coronary perfusion [29]. Volume overload, common in PD, further elevates PP by increasing stroke volume [30]. In the present cohort, patients in the highest PP tertile had higher serum uric acid and Hs-CRP. Prior evidence links uric acid to endothelial dysfunction, oxidative stress, and vascular calcification [31], and systemic inflammation to arterial stiffness [32]; these observations support a biologically plausible hypothesis—rather than an established causal pathway—that uric acid and inflammation may contribute to arterial stiffening in PD. We acknowledge that volume overload may simultaneously influence SBP, stroke volume, PP, and mortality; because objective volume measures were not available in this cohort, residual confounding by volume status represents an important limitation to be addressed in future studies.

Only 9.7% of participants achieved the nocturnal BP goal. The independent association between 24 h PP and adverse outcomes after adjustment for office PP suggests that ABPM-derived PP may serve as a candidate marker for CV risk stratification in PD patients. Nevertheless, the present observational data do not demonstrate that routine ABPM-guided PP management improves outcomes. Future multicenter prospective studies should validate clinically useful PP thresholds, assess repeated ABPM measurements and longitudinal changes in 24 h PP, incorporate direct measures of arterial stiffness (e.g., carotid–femoral PWV) and objective volume-status assessment, and determine whether 24 h PP adds discrimination beyond established CV risk factors. Whether interventions targeting volume overload, BP control, or CKD–MBD modify the risk associated with elevated PP also warrants investigation.

Several limitations warrant acknowledgement. First, this was a single-center cohort with limited events (28 CV deaths; approximately four events per variable), which constrained the precision of categorical estimates and precluded formal assessment of incremental predictive performance, although results were directionally consistent across parsimonious, cause-specific Cox, and bootstrap sensitivity analyses. Second, selection bias may exist: ABPM participation was voluntary among incident PD patients, and the minimum 3-month PD requirement may have excluded early deaths. Third, exposures and confounders were incompletely measured: ABPM and PP were assessed only at baseline; subsequent changes in BP treatment, volume status, or PP during follow-up may therefore introduce exposure misclassification; objective volume status, dialysis adequacy, PD modality, residual urine output, medication dosing and adherence, and CKD–MBD parameters were unavailable; and direct stiffness measures such as PWV were not performed. Fourth, 34 patients (20.6%) transferred to HD or underwent transplantation and were treated as censored, so informative (non-independent) censoring cannot be fully excluded, and out-of-hospital cause-of-death assignment may be misclassified. Finally, the relatively young age and low diabetes prevalence may limit generalizability.

## 5. Conclusions

Elevated ABPM-derived 24 h PP was independently associated with higher all-cause and cardiovascular mortality in this single-center cohort of PD patients, including after adjustment for office PP and selected conventional risk factors. These findings suggest that 24 h PP may provide complementary information for CV risk stratification. Larger multicenter prospective studies incorporating repeated ABPM, direct measures of arterial stiffness and volume status, and external validation are required before 24 h PP can be incorporated into routine risk-stratification or treatment algorithms.

## Figures and Tables

**Figure 1:**
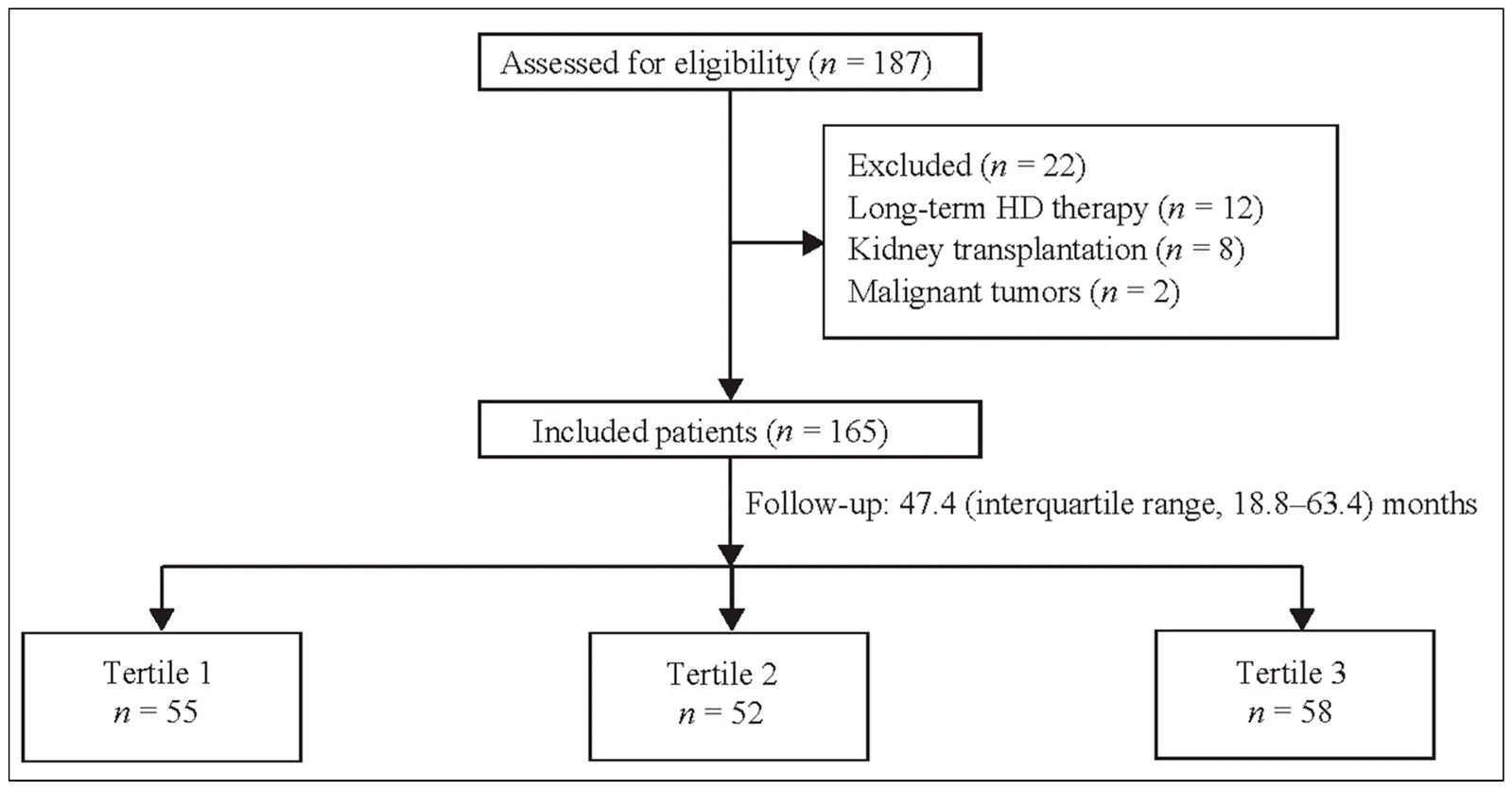
Patient flow. Among 565 incident PD patients managed at our center during the recruitment period, 187 consented to undergo ABPM and were initially assessed. Following exclusion of 22 patients who did not meet eligibility criteria, 165 patients were retained for the final analytical cohort. Abbreviations: HD, hemodialysis.

**Figure 2:**
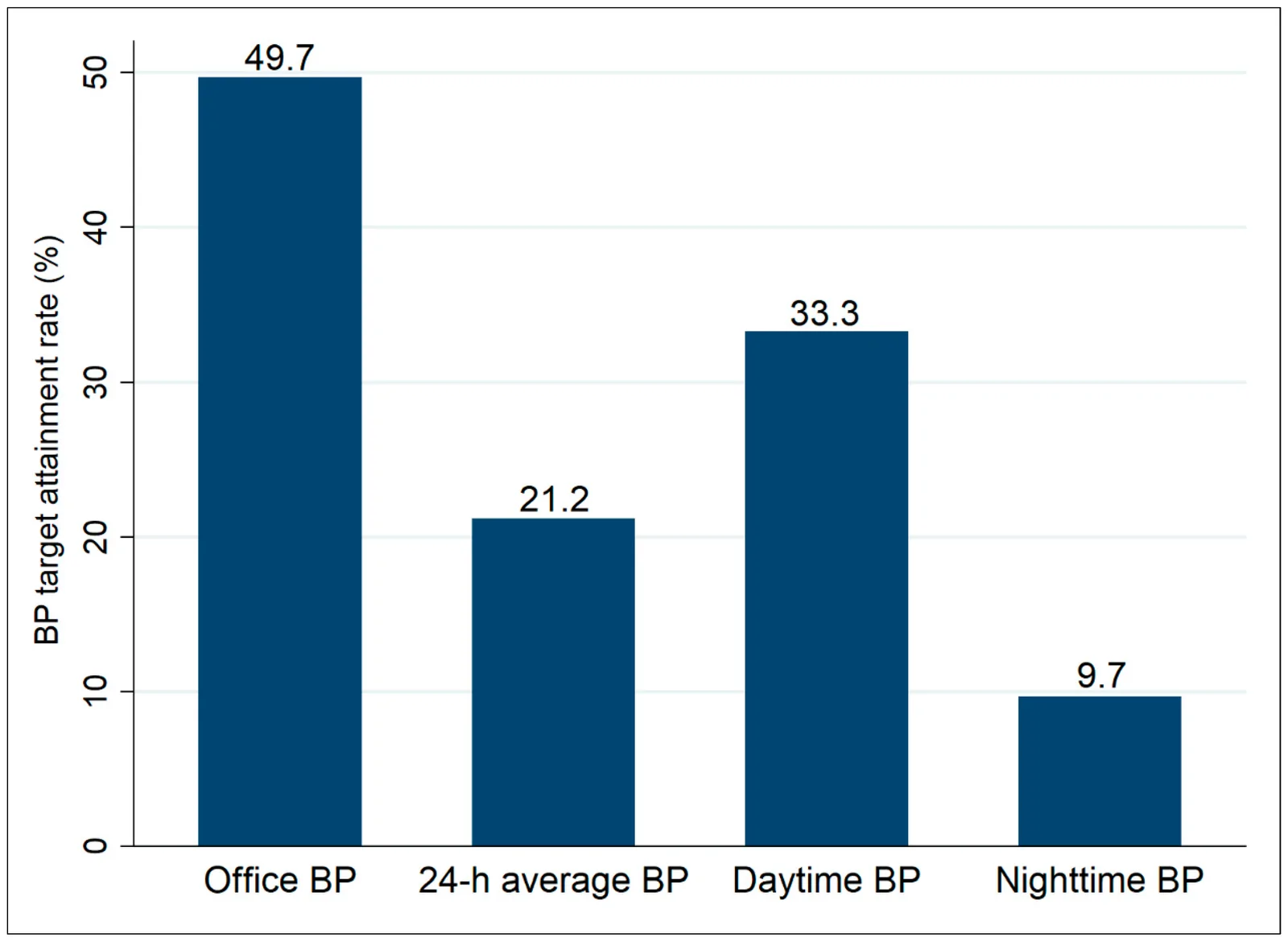
Proportion of patients achieving BP control targets according to four measurement modalities. Office BP target was defined as <140/90 mmHg; 24 h average BP target as <130/80 mmHg; daytime BP target as <135/85 mmHg; and nighttime BP target as <120/70 mmHg. Abbreviations: BP, blood pressure.

**Figure 3:**
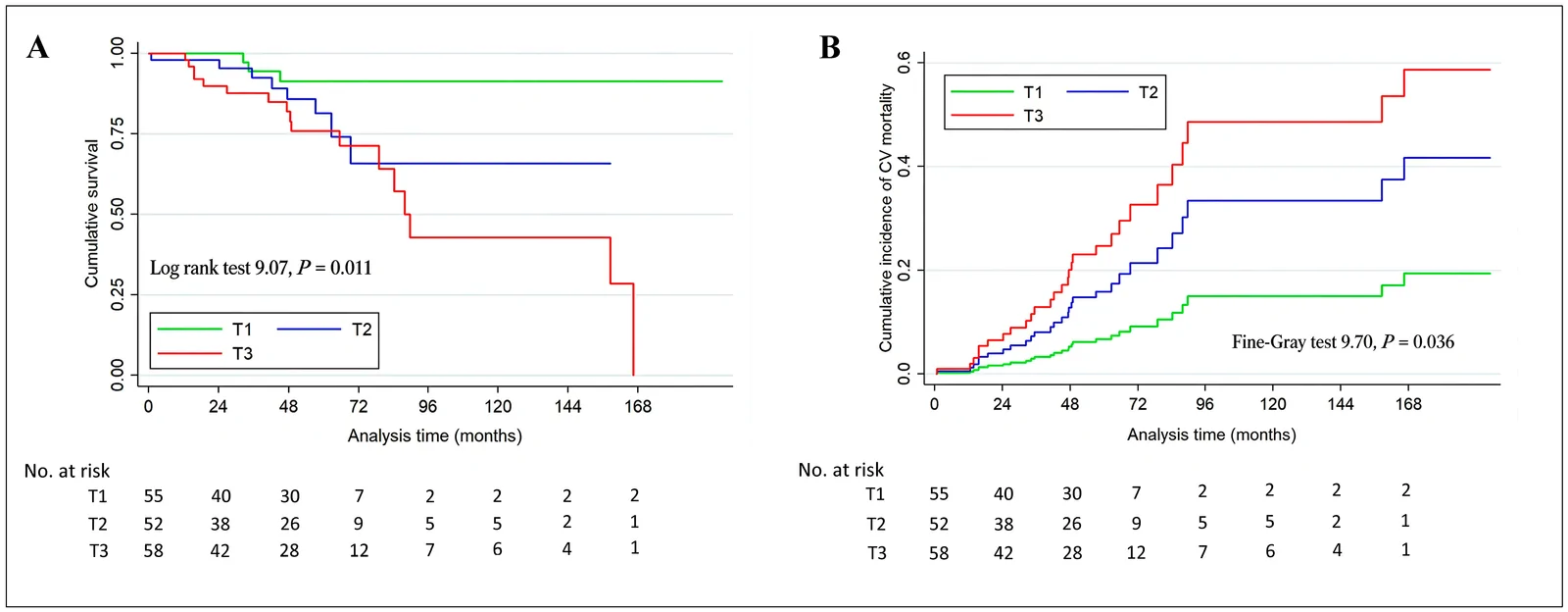
Survival estimates according to 24 h PP tertiles. (**A**) Kaplan–Meier curves for cumulative all-cause survival across T1, T2, and T3 (log-rank *p* = 0.011). (**B**) Cumulative incidence function curves for CV mortality, with non-CV death treated as a competing event (Gray’s test *p* = 0.036). Abbreviations: CV, cardiovascular; T1–T3, lowest to highest tertile; PP, pulse pressure.

**Figure 4:**
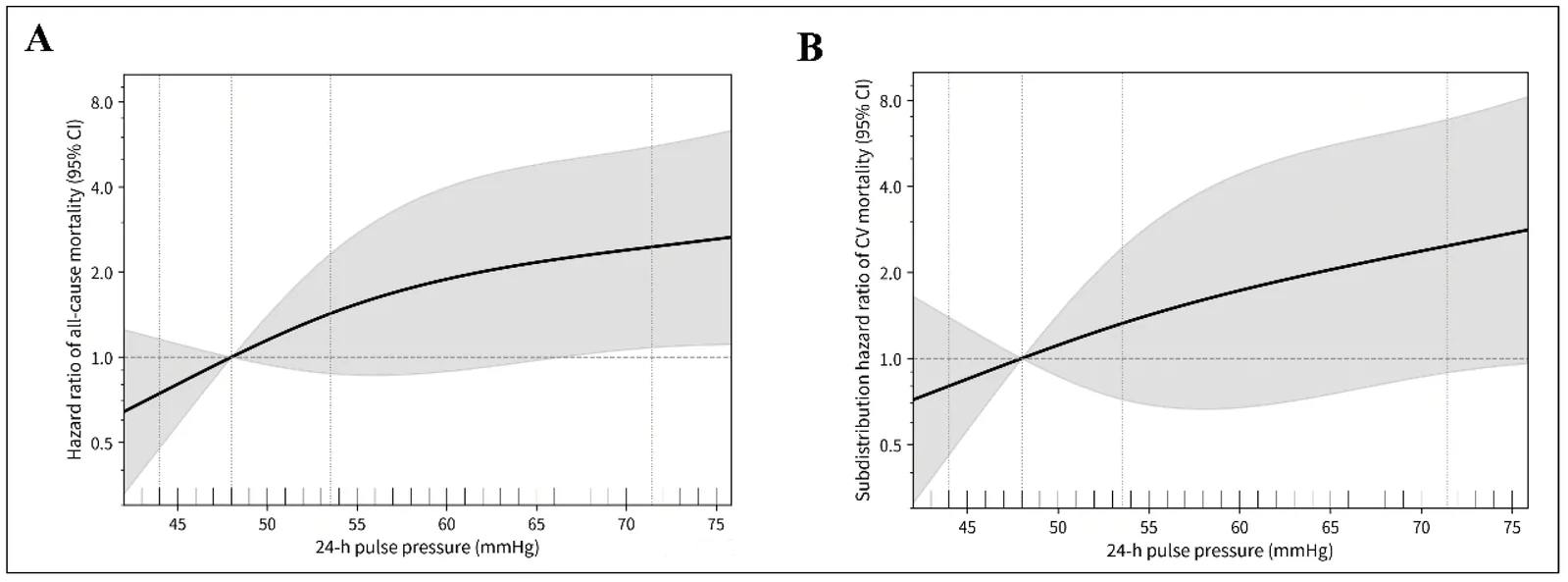
Restricted cubic spline analyses of the dose–response relationship between 24 h PP and mortality. Splines were fitted with three knots placed at the 10th, 50th, and 90th percentiles of 24 h PP (44, 54, and 71 mmHg), adjusted for age, sex, diabetes, prior CV events, serum albumin, uric acid, and office PP. The *x*-axis displays the 5th–95th percentile range (42–76 mmHg), with 48 mmHg (the upper boundary of the lowest tertile) as the reference. The rug plot at the bottom shows the distribution of individual observations. Shaded areas represent 95% confidence intervals. (**A**) Hazard ratio of all-cause mortality; (**B**) Subdistribution hazard ratio of CV mortality. Abbreviations: CI, confidence interval; CV, cardiovascular.

**Table 1:** Baseline characteristics of the study cohort.

Covariates	Total (*n* = 165)	Tertiles of 24 h PP (mmHg)			*p* Value
		T1 (<48) (*n* = 55)	T2 (48–54) (*n* = 52)	T3 (>54) (*n* = 58)	
Age (years)	42.4 ± 13.4	37.9 ± 11.3	41.5 ± 13.0	47.5 ± 14.1	<0.001
Male *n* (%)	96 (58.2)	29 (52.7)	27 (51.9)	40 (69.0)	0.118
Smoking *n* (%)	44 (26.7)	11 (20.0)	12 (23.1)	21 (36.2)	0.117
Diabetes *n* (%)	18 (10.9)	2 (3.6)	4 (7.7)	12 (20.7)	0.009
Prior CV events *n* (%)	16 (9.7)	6 (10.9)	5 (9.6)	5 (8.6)	0.919
Hypertension *n* (%)	156 (94.5)	50 (90.9)	51 (98.1)	55 (94.8)	0.262
BMI (kg/m^2^)	21.9 ± 3.1	20.6 ± 2.7	21.9 ± 3.4	23.0 ± 2.9	<0.001
Hemoglobin (g/L)	90.0 ± 20.0	94.7 ± 22.5	88.3 ± 19.6	85.8 ± 16.7	0.050
Serum albumin (g/L)	32.9 ± 4.7	33.9 ± 5.1	32.4 ± 4.7	32.3 ± 4.1	0.121
Hs-CRP (mg/L)	2.25 [0.78–7.05]	1.50 [0.72–3.30]	2.88 [1.03–14.11]	2.93 [0.80–9.25]	0.041
TC (mg/dL)	172.8 ± 51.5	180.1 ± 50.5	158.9 ± 42.4	178.4 ± 57.9	0.061
TG (mg/dL)	121.2 [92.0–169.0]	131.0 [93.8–167.3]	117.7 [90.3–150.4]	116.4 [88.5–183.6]	0.508
HDL-C (mg/dL)	43.0 ± 13.4	46.3 ± 15.2	40.1 ± 9.3	42.6 ± 14.2	0.055
LDL-C (mg/dL)	105.3 ± 35.8	108.7 ± 34.2	96.1 ± 32.0	110.4 ± 39.5	0.078
Uric acid (mmol/L)	437.3 ± 119.5	430.2 ± 115.4	428.2 ± 134.5	452.0 ± 109.2	0.505
eGFR (mL/min/1.73 m^2^)	5.5 ± 2.1	6.3 ± 2.4	5.1 ± 1.9	5.1 ± 1.7	0.002
Office SBP (mmHg)	138.8 ± 20.6	125.6 ± 15.1	139.7 ± 17.4	150.7 ± 20.5	<0.001
Office DBP (mmHg)	86.0 ± 14.1	81.3 ± 10.5	87.8 ± 14.9	88.9 ± 15.2	0.008
Office PP (mmHg)	52.8 ± 14.8	44.3 ± 13.3	51.8 ± 9.0	61.8 ± 15.6	<0.001
24 h SBP (mmHg)	140.0 ± 16.4	125.0 ± 10.6	139.9 ± 10.6	153.4 ± 13.0	<0.001
24 h DBP (mmHg)	84.2 ± 10.3	79.7 ± 10.3	86.3 ± 9.8	86.4 ± 9.3	<0.001
24 h PP (mmHg)	55.5 ± 10.6	45.3 ± 3.2	53.6 ± 2.3	67.0 ± 8.4	<0.001
Daytime SBP (mmHg)	140.5 ± 16.8	125.8 ± 10.6	140.3 ± 10.8	154.6 ± 13.7	<0.001
Daytime DBP (mmHg)	84.9 ± 10.4	80.7 ± 10.4	86.8 ± 9.9	87.3 ± 9.8	0.001
Nighttime SBP (mmHg)	137.8 ± 18.1	122.9 ± 13.7	139.1 ± 14.1	150.8 ± 14.0	<0.001
Nighttime DBP (mmHg)	82.5 ± 11.5	77.4 ± 11.4	85.5 ± 11.7	84.6 ± 9.8	<0.001
Antihypertensive drugs *n* (%)	153 (92.7)	48 (87.3)	51 (98.1)	54 (93.1)	0.137
ACEI/ARB, *n* (%)	103 (62.4)	30 (54.5)	36 (69.2)	37 (63.8)	0.282
CCB, *n* (%)	125 (75.8)	36 (65.5)	41 (78.8)	48 (82.8)	0.082
β-blocker, *n* (%)	95 (57.6)	28 (50.9)	32 (61.5)	35 (60.3)	0.468
α-blocker, *n* (%)	30 (18.2)	4 (7.3)	9 (17.3)	17 (29.3)	0.010
Diuretic, *n* (%)	23 (13.9)	6 (10.9)	4 (7.7)	13 (22.4)	0.061
No. of drug classes	2 [1–3]	2 [1–3]	2 [2–3]	3 [2–3]	0.006
Statin use *n* (%)	34 (20.6)	10 (18.2)	9 (17.3)	15 (25.9)	0.467

Notes: Normally distributed data were expressed as mean ± standard deviation (SD), whereas non-normal data were presented as median (interquartile range, IQR). The distribution of categorical variables was displayed via absolute frequencies and relative percentages. Abbreviations: ACEI, angiotensin-converting enzyme inhibitors; ARB, angiotensin II receptor antagonists; BMI, body mass index; CCB, calcium channel blockers; CV, cardiovascular; DBP, diastolic blood pressure; eGFR, estimated glomerular filtration rate; HDL-C, high-density lipoprotein cholesterol; Hs-CRP, hypersensitive C-reactive protein; LDL-C, low-density lipoprotein cholesterol; PP, pulse pressure; SBP, systolic blood pressure; TC, total cholesterol; TG, triglycerides; T1–T3, lowest to highest tertile.

**Table 2:** Independent associated factors for 24 h PP.

Associated Factors	Univariate Logistic Regression		Multivariate Logistic Regression	
	Crude OR (95% CI)	*p* Value	Adjusted OR (95% CI)	*p* Value
Age (per 1-year greater)	1.08 (1.04–1.11)	<0.001	1.08 (1.03–1.12)	<0.001
Sex (female/male)	0.71 (0.32–1.55)	0.388		
Smoking (yes/no)	1.42 (0.63–3.22)	0.401		
Diabetes (yes/no)	6.35 (2.27–17.76)	<0.001	4.26 (1.37–13.20)	0.012
Prior CV events (yes/no)	1.32 (0.40–4.39)	0.648		
BMI (per 1 kg/m^2^ greater)	1.14 (1.01–1.29)	0.033		
Hemoglobin (per 1 g/L greater)	0.99 (0.98–1.01)	0.577		
Serum albumin (per 1 g/L greater)	0.96 (0.88–1.04)	0.291		
Hs-CRP (per 1 mg/L greater)	1.00 (0.96–1.03)	0.850		
Uric acid (per 1-SD greater)	1.13 (0.77–1.65)	0.531	1.72 (1.06–2.79)	0.028
eGFR (per 1 mL/min/1.73 m^2^ greater)	0.83 (0.68–1.03)	0.084		

Note: Higher 24 h PP levels refer to tertile 3. The effective sample size was 165 for the multivariable logistic regression model, with no missing values observed for all model covariates. Abbreviations: BMI, body mass index; CI, confidence interval; CV, cardiovascular; eGFR, estimated glomerular filtration rate; Hs-CRP, hypersensitive C-reactive protein; OR, odds ratio; PP, pulse pressure; SD, standard deviation.

**Table 3:** Association of 24 h PP with all-cause and CV mortality.

	Per 1-SD Increase		T2 (*n* = 52)		T3 (*n* = 58)	
	HR (95% CI)	*p* Value	HR (95% CI)	*p* Value	HR (95% CI)	*p* Value
All-cause mortality						
Unadjusted	1.41 (1.12–1.78)	0.004	2.97 (1.07–8.24)	0.037	3.98 (1.52–10.42)	0.005
Model 1	1.30 (1.00–1.69)	0.047	2.65 (0.94–7.44)	0.065	3.11 (1.12–8.61)	0.029
Model 2	1.38 (1.06–1.81)	0.018	2.41 (0.85–6.85)	0.097	3.43 (1.22–9.66)	0.020
Model 3	1.39 (1.03–1.87)	0.032	2.42 (0.84–6.97)	0.100	3.47 (1.12–10.70)	0.031
	SHR (95% CI)	*p* Value	SHR (95% CI)	*p* Value	SHR (95% CI)	*p* Value
CV mortality						
Unadjusted	1.47 (1.08–1.99)	0.014	2.51 (0.65–9.64)	0.181	4.10 (1.21–13.90)	0.023
Model 1	1.23 (0.89–1.68)	0.205	1.85 (0.44–7.75)	0.401	2.78 (0.73–10.57)	0.133
Model 2	1.27 (0.94–1.72)	0.118	1.81 (0.44–7.54)	0.413	3.18 (0.89–11.35)	0.075
Model 3	1.44 (1.06–1.94)	0.018	2.17 (0.51–9.20)	0.293	5.11 (1.32–19.79)	0.018

Notes: We indicated the lowest tertile (T1) as the reference group (*n* = 55). All multivariable models (Models 1–3) comprised 165 participants. No missing values were found among covariates entered into the models, and missing data were managed via complete-case analysis. Model 1: Adjusted for age and sex (*n* = 165). Model 2: Adjusted for model 1 plus diabetes, prior CV events, serum albumin, and uric acid (*n* = 165). Model 3: Adjusted for model 2 plus office PP (*n* = 165). Abbreviations: CI, confidence interval; CV, cardiovascular; HR, hazard ratio; PP, pulse pressure; SD, standard deviation; SHR, subdistribution hazard ratio; T1 to T3, lowest to highest tertile.

## Data Availability

The data presented in this study are available on request from the corresponding author. The data are not publicly available due to patient-privacy and ethical restrictions.
